# Evaluation of Aminopolycarboxylate Chelators for Whole-Body Clearance of Free ^225^Ac: A Feasibility Study to Reduce Unexpected Radiation Exposure during Targeted Alpha Therapy

**DOI:** 10.3390/pharmaceutics13101706

**Published:** 2021-10-16

**Authors:** Mitsuyoshi Yoshimoto, Yukie Yoshii, Hiroki Matsumoto, Mitsuhiro Shinada, Masashi Takahashi, Chika Igarashi, Fukiko Hihara, Tomoko Tachibana, Ayano Doi, Tatsuya Higashi, Hirofumi Fujii, Kohshin Washiyama

**Affiliations:** 1 Division of Functional Imaging, National Cancer Center Hospital East, Kashiwa 277-8577, Japan; miyoshim@ncc.go.jp (M.Y.); aydoi@east.ncc.go.jp (A.D.); hifujii@east.ncc.go.jp (H.F.); 2 National Institutes for Quantum and Radiological Science and Technology, Chiba 263-8555, Japan; matsumoto.hiroki2@qst.go.jp (H.M.); 3052shinada.mitsuhiro2016@gmail.com (M.S.); marshi55@mbc.nifty.com (M.T.); igarashi.chika@qst.go.jp (C.I.); hihara.fukiko@qst.go.jp (F.H.); tachibana.tomoko@qst.go.jp (T.T.); higashi.tatsuya@qst.go.jp (T.H.); 3 Faculty of Science, Toho University, Funabashi 274-8510, Japan; 4 Advanced Clinical Research Center, Fukushima Medical University, Fukushima 960-1295, Japan; kwashi@fmu.ac.jp

**Keywords:** aminopolycarboxylate chelators, free ^225^Ac, targeted alpha therapy, unexpected radiation exposure

## Abstract

Actinium-225 (^225^Ac) is a promising radionuclide used in targeted alpha therapy (TAT). Although ^225^Ac labeling of bifunctional chelating ligands is effective, previous in vivo studies reported that free ^225^Ac can be released from the drugs and that such free ^225^Ac is predominantly accumulated in the liver and could cause unexpected toxicity. To accelerate the clinical development of ^225^Ac TAT with a variety of drugs, preparing methods to deal with any unexpected toxicity would be valuable. The aim of this study was to evaluate the feasibility of various chelators for reducing and excreting free ^225^Ac and compare their chemical structures. Nine candidate chelators (D-penicillamine, dimercaprol, Ca-DTPA, Ca-EDTA, CyDTA, GEDTA TTHA, Ca-TTHA, and DO3A) were evaluated in vitro and in vivo. The biodistribution and dosimetry of free ^225^Ac were examined in mice before an in vivo chelating study. The liver exhibited pronounced ^225^Ac uptake, with an estimated human absorbed dose of 4.76 Sv_RBE5_/MBq. Aminopolycarboxylate chelators with five and six carboxylic groups, Ca-DTPA and Ca-TTHA, significantly reduced ^225^Ac retention in the liver (22% and 30%, respectively). Significant ^225^Ac reductions were observed in the heart and remainder of the body with both Ca-DTPA and Ca-TTHA, and in the lung, kidney, and spleen with Ca-TTHA. In vitro interaction analysis supported the in vivo reduction ability of Ca-DTPA and Ca-TTHA. In conclusion, aminopolycarboxylate chelators with five and six carboxylic groups, Ca-DTPA and Ca-TTHA, were effective for whole-body clearance of free ^225^Ac. This feasibility study provides useful information for reducing undesirable radiation exposure from free ^225^Ac.

## 1. Introduction

Actinium-225 (^225^Ac) is a promising α-particle-emitting radionuclide used in targeted alpha therapy (TAT) [1,2]. ^225^Ac (*T*_1/2_ = 9.92 d) generates short-lived daughter nuclides and emits four high-linear energy transfer α particles in total, until a long half-life of ^209^Bi (2.01 × 10^21^ y) is reached. This induces lethal damage to target cancer cells in ^225^Ac TAT [1,2]. High-quality ^225^Ac is easily produced from ^229^Th generators (*T*_1/2_ = 7.3 y). ^225^Ac is compatible with various targeting agents, such as peptides, antibodies, and nanoparticles, and ^225^Ac-labeled drugs exhibit great efficacy in vivo [1,2]. ^225^Ac-PSMA-617 is a promising agent for prostate cancer metastasis, as reported in preliminary clinical studies [3], and is currently under phase 1 clinical trials (AcTION: NCT04597411). Various ^225^Ac-labeled antibodies have also been developed in clinical trials, such as ^225^Ac-FPI-1434 and ^225^Ac-lintuzumab (NCT03867682) [4,5]. Currently, many other clinical trials, using a variety of ^225^Ac-labeled drugs targeting different types of cancers, are underway; therefore, ^225^Ac TAT is expected to provide new therapeutic opportunities to cancer patients in the near future [6].

Numerous bifunctional chelating ligands for the ^225^Ac-labeling of drugs have been evaluated [7,8]. DOTA is the most commonly used bifunctional chelating ligand in ^225^Ac-labeled drugs and has been transferred to recent clinical trials [7,8]. Although bifunctional chelating ligands are highly useful and effective for ^225^Ac-labeling, previous in vivo studies with a variety of drugs also reported that free ^225^Ac can be released from TAT drugs, and that such free ^225^Ac could cause unexpected toxicity [1,7,8,9,10,11,12,13]. Hence, to accelerate the future clinical development of ^225^Ac TAT with various drugs, it would be valuable to prepare methods to deal with any unexpected toxicity. Free ^225^Ac is reported to be distributed predominantly in the liver, followed by the bones [1,7,8,9,10,11,12,13]. A previous study reported that free ^225^Ac administration causes morphological changes in mouse livers, such as diffuse hepatocellular cytoplasmic vacuolation, consistent with glycogen accumulation or hydropic degeneration [10]. Free ^225^Ac administration does not cause morphological changes in the bones; i.e., the bone marrow contains cellular progenitors from myeloid, erythroid, and megakaryocyte, similarly to controls, although a transient decrease in white blood cells is observed [10]. This might be because α particles emitted from radionuclides that accumulate on the bone surface have little effect on the bone marrow, owing to their short range [14,15]. Therefore, the liver is considered the major critical organ for free ^225^Ac, and it is necessary to develop methods for reducing and excreting free ^225^Ac from the liver.

**Figure 1:**
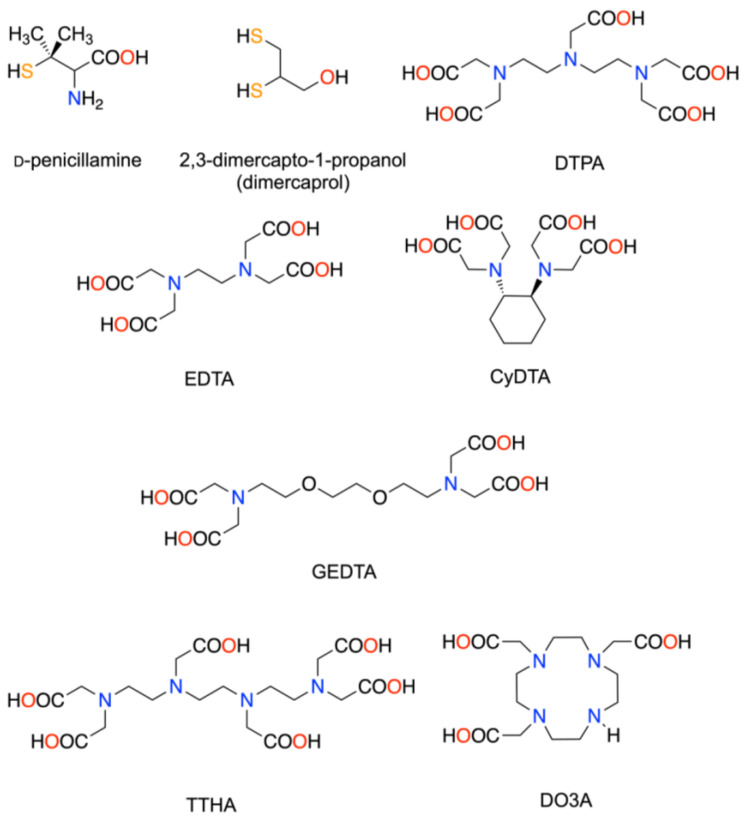
Chemical structures of the candidate chelators. Possible ligating atoms are colored.

Several chelator drugs are used clinically to reduce the toxicity caused by the non-radioactive and radioactive heavy metals that are unexpectedly accumulated in the body. Nine candidate chelators were investigated in this study, namely D-penicillamine, dimercaprol, Ca-DTPA, Ca-EDTA, CyDTA, GEDTA, TTHA, Ca-TTHA, and DO3A (the chemical names are shown in Table 1) (Figure 1). Penicillamine is used to reduce copper accumulation in Wilson’s disease [16]. Dimercaprol is indicated for the treatment of arsenic, gold, and mercury poisoning [17]. Ca-EDTA is indicated for treating acute lead poisoning [18]. Ca-DTPA is used for treating internal contamination with plutonium, americium, or curium [19]. We hypothesized that the aforementioned chelators may also reduce free ^225^Ac released from drugs during ^225^Ac TAT and accumulated in the body. CyDTA, GEDTA, and TTHA are commercially available aminopolycarboxylate chelators that form a stable complex with a lanthanum (III) ion (La^3+^), which has a similar chemical nature (3+ charge and closed subshell electron configuration) and an ionic radius close to actinium (Ac^3+^) [20,21,22]. Ca-TTHA was included in our study to avoid potential Ca depletion in the body by TTHA. DO3A is a macrocyclic chelator that forms a stable complex with gadolinium and is used as a contrast agent for magnetic resonance imaging in clinical practice [23]. DOTA was not used in this study, since the labeling of biomolecules with ^225^Ac is conventionally performed using DOTA [24,25], and an excess amount of DOTA in the blood can interfere with ^225^Ac-DOTA-drugs. Moreover, DOTA and other macrocyclic aminopolycarboxylates were not considered from a kinetic viewpoint; e.g., cyclic chelators require a longer time than acyclic chelators to form thermodynamically stable complexes [26]. This kinetic inertness would be a disadvantage in a flow system such as the bloodstream.

In this study, we examined the interaction and effects of the nine chelators with ^225^Ac in vitro and on the biodistribution of free ^225^Ac in mice, to evaluate the feasibility of these chelators for reducing and excreting free ^225^Ac. We also assessed the association between the reduction in ^225^Ac retention and the chemical structure of the chelators.

## 2. Materials and Methods

### 2.1. Radionuclides

^225^Ac (*T*_1/2_ = 9.92 d) was obtained from a ^229^Th (*T*_1/2_ = 7880 y) stock solution provided by the Laboratory of Alpha-Ray Emitters, Institute for Materials Research, Tohoku University. In the ^229^Th stock solution, ^225^Ac had reached a sufficient equilibrium state with its parent nuclides, ^229^Th and ^225^Ra (*T*_1/2_ = 14.9 d). ^225^Ac was separated from ^229^Th using a previously reported method [27], with slight modifications. Briefly, ^225^Ac was separated from the ^229^Th solution (5 mL) prepared in 7M HNO_3_ passing through a 5 × 42 mm^2^ column containing Muromac AG1 × 8 anion exchange resin (Muromachi Technos Co., Ltd., Tokyo, Japan) pre-equilibrated with 7 M HNO_3_. The column was washed with 15 mL 7 M HNO_3_ to collect trace amounts of ^225^Ac. The eluate was diluted to 4M HNO_3_ and purified by separation of ^229^Th and ^225^Ra, using a tandem combination of UTEVA Resin and DGA Resin (Eichrom Technologies, LLC, Lisle, IL, USA). In this system, ^225^Ra was passed through all cartridge systems, whereas trace amounts of ^229^Th and the desired ^225^Ac were retained by UTEVA Resin and DGA Resin, respectively. ^225^Ac was then recovered from the DGA Resin using 10 mL 0.05 M HNO_3_. The eluted ^225^Ac solution was evaporated almost to dryness, and then re-dissolved in 0.1 M HCl as a stock solution. The ^225^Ac solution was adjusted to neutral pH using 3 M ammonium acetate buffer (pH 6.0); the solution was then diluted to a concentration of 10 kBq/100 µL with saline for in vitro and in vivo studies. The radioactivity of ^225^Ac was quantified using a germanium semiconductor detector (ORTEC, SEIKO EG&G, Tokyo, Japan). Following ^225^Ac separation, ^229^Th was recovered from UTEVA Resin with 0.5 M HCl for ^225^Ac ingrowth.

### 2.2. Preparation of Reagents

The chelators used in this study are listed in Table 1. Ca-DTPA and D-penicillamine were dissolved in sterile water. Ca-EDTA, GEDTA, CyDTA, TTHA, and DO3A were dissolved in sterile water with an aliquot of sodium hydroxide, and the pH was adjusted between 8–9, if necessary. Ca-TTHA was prepared by adding an equal amount of calcium dichloride to TTHA in sterile water, and the pH was adjusted between 8–9 using a sodium hydroxide solution. Under this condition, the quantitative coordination of DTPA, EDTA, and TTHA to Ca^2+^ in the sterile water was confirmed by calculating the dissolved species. Dimercaprol was diluted with peanut oil (Nacalai Tesque, Kyoto, Japan).

### 2.3. In Vitro Analysis of ^225^Ac Chelates Formation

^225^Ac solution (20 μL; 10 kBq/100 µL) was mixed with 20 µL chelator solution (Table 1) and incubated at 37 °C for 30 min. A 1 μL aliquot of each sample was applied to chromatography papers (3MM, Whatman, Little Chalfont, UK) and developed with 0.9% NaCl as the mobile phase. This system is reported to separate polar ^225^Ac-chelator complexes from unbound ^225^Ac, and the ^225^Ac chelate was observed at the solvent front, and free ^225^Ac was observed at the origin [28]. The samples were left overnight to allow for the decay of daughter nuclides and for attaining a secular equilibrium of ^225^Ac; the radioactivity from daughter nuclides of ^225^Ac, i.e., gamma rays from ^221^Fr (218 keV) and ^213^Bi (440 keV), was analyzed using a bioimaging analyzer (FLA-7000, GE Healthcare, Chicago, IL, USA).

### 2.4. Animals

BALB/cAnNCrlCrj female mice (6 weeks old, 18–22 g body weight) were obtained from Charles River Laboratories. The mice were weighed and randomized for analysis after more than seven days of acclimatization. All animal experiments were approved by the Animal Ethics Committee of our institution and conducted in accordance with institutional guidelines (National Institutes for Quantum and Radiological Science and Technology, approval no. 13-1022-7).

### 2.5. Biodistribution of Free ^225^Ac in Mice and Dosimetry Analysis

Biodistribution was studied in four mice per group at 5 min, 1 h, 4 h, 24 h, and 72 h after the injection with free ^225^Ac. Animals in the 5 min to 4 h sampling groups were housed individually, and the urine and feces were collected with polyethylene-laminated filter papers. Animals in the 24 h and 72 h sampling groups were housed individually in metabolic cages (3600M021; Tecniplast S.p.A, Buguggiate, Italy) to collect urine and feces. The organs of the mice, such as the salivary glands, heart, lung, liver, kidney, spleen, pancreas, brain, muscle, and femur, along with the remainder of the body and the blood were collected and weighed. The samples were left overnight to allow for the decay of daughter nuclides and attaining a secular equilibrium of ^225^Ac, and the radioactivity from daughter nuclides of ^225^Ac, and gamma rays from ^221^Fr (218 keV) were measured using a γ-counter (2480 Wizard 2; PerkinElmer, Waltham, MA, USA). The biodistribution data were calculated and are shown as the % of injected dose (ID)/g for the organs and blood and the % ID for the urine and feces. The mean absorbed doses of ^225^Ac (mSv/MBq) in humans were estimated based on biodistribution data. The mean % ID/g values of the mouse organs were converted into the corresponding human values [29]. These values were processed with OLINDA/EXM version 1.1 software [30], which used a relative biological efficacy (RBE) for α particles of 5 and a dynamic bladder model with a voiding interval of 4.8 h to estimate the organ doses (Sv _RBE5_/MBq). Considering instant decay of the daughter nuclides of ^225^Ac (^221^Fr, ^217^At, ^213^Bi, ^213^Po, ^209^Tl, and ^209^Pb) without translocation during the decay, the residency times of ^225^Ac were forwarded as those of daughter nuclides [3].

### 2.6. In Vivo Chelating Study

**Figure 2:**
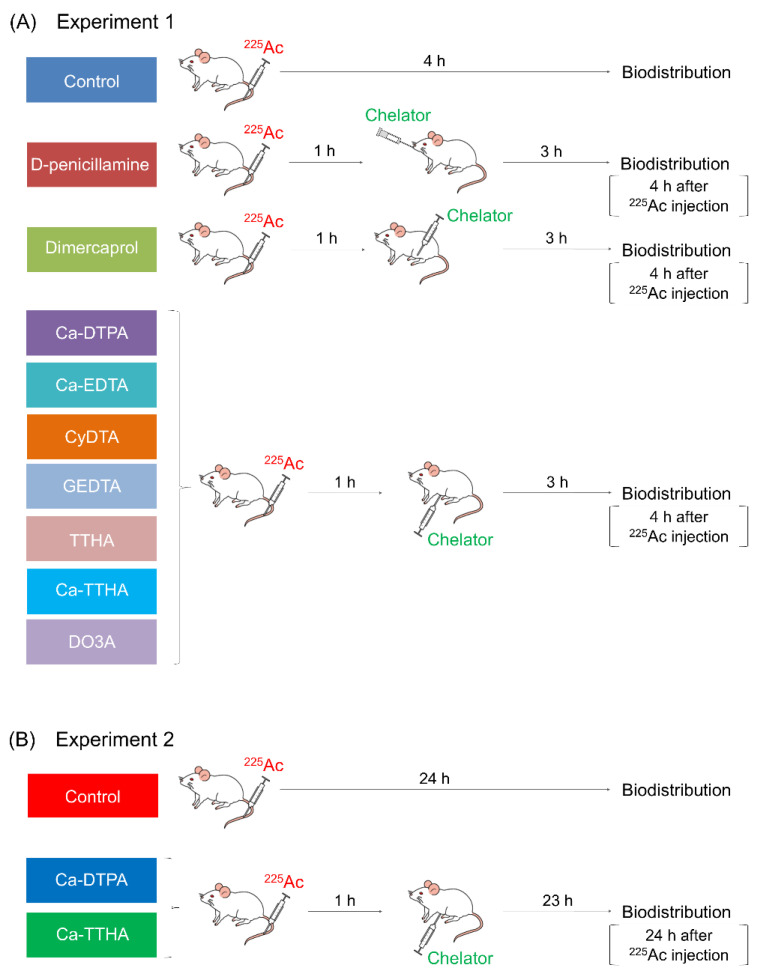
Schedule of the in vivo chelating study. Biodistribution studies of free ^225^Ac with chelators as conducted in Experiments 1 (**A**) and 2 (**B**).

The schedule of the in vivo chelating study is summarized in Figure 2.

#### 2.6.1. Experiment 1

^225^Ac (10 kBq/mouse) was intravenously injected into the mice. At 1 h post ^225^Ac injection, the mice were treated with each chelator solution, except for the untreated controls. In total, five mice were used in each chelator solution group, except for the Ca-TTHA group, which included four mice. The administered doses and routes are summarized in Table 1 and were determined as follows: Dimercaprol was administered intramuscularly (i.m.) to the mice, and the dose was approximately half of the LD_50_ in mice, or 125 mg/kg [31]; D-penicillamine was administered orally (p.o.) to the mice, and the dose was determined according to our previous study with ^64^Cu-ATSM and D-penicillamine [32]. Ca-DTPA and the remaining compounds were intraperitoneally (i.p.) administered, and their doses were sufficiently lower than the LD_50_ of Ca-DTPA in mice (6216.9 mg/kg) [33]. The mice were housed individually, and urine and feces were collected using polyethylene-laminated filter papers. At 4 h after ^225^Ac administration, the animals were euthanized, and biodistributions were evaluated as described in Section 2.5.

#### 2.6.2. Experiment 2

Ca-DTPA and Ca-THHA showed the highest reduction in the rate of liver uptake among the chelators tested; therefore, their effects were observed over a longer time period. At 1 h following ^225^Ac injection (10 kBq/mouse), the mice were administered solutions of Ca-DTPA or Ca-TTHA, except for the untreated controls. A total of four animals were included in each chelator solution group. At 24 h following ^225^Ac administration, the mice were euthanized, and the free ^225^Ac biodistributions were evaluated as described in Section 2.5, as 24 h is the peak time point of biodistribution observation for the liver. The time-activity curves were created using the biodistribution data at 4 h and 24 h, to compare the effects of these chelators with the control.

### 2.7. Statistical Analysis

Data are expressed as means ± SD. Multiple comparisons were conducted using one-way analysis of variance (ANOVA) or a Kruskal–Wallis test, with post hoc comparisons using Tukey–Kramer or Steel–Dwass tests. Time–activity curves were compared using a two-way repeated ANOVA. Data analyses were conducted using JMP 13.2.0 software (SAS Institute). *p* < 0.05 was considered statistically significant.

## 3. Results

### 3.1. In Vitro Analysis of ^225^Ac Chelates Formation

**Figure 3:**
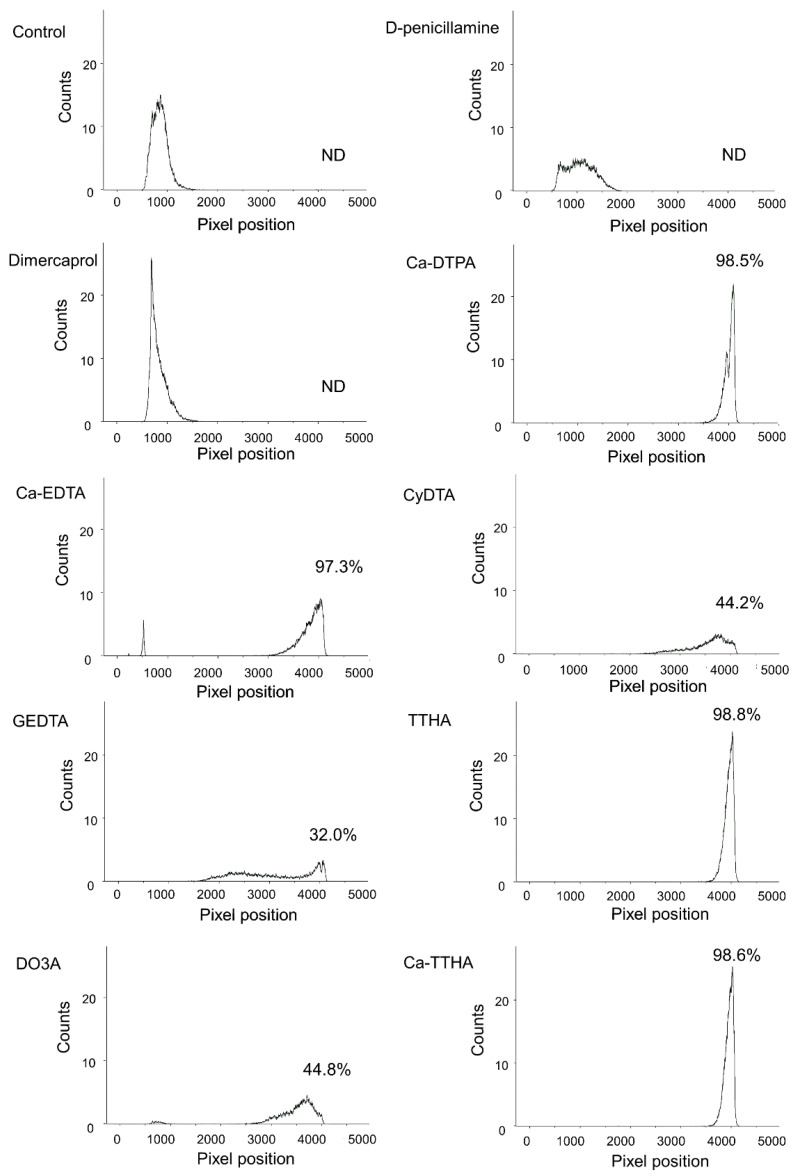
In vitro ability of the chelators to capture actinium-225 (^225^Ac). Paper chromatography of free ^225^Ac alone (control) and free ^225^Ac with chelators. The percentages of radioactivity at the solvent front (^225^Ac chelates) are shown for each chromatograph. ND = not detected.

The ability of the chelators to capture ^225^Ac in vitro is shown in Figure 3. Free ^225^Ac (control) remained at its origin. In contrast, the ^225^Ac with Ca-DTPA, Ca-EDTA, Ca-TTHA, and TTHA developed greatly, with more than 95% of the radioactivity at the solvent front. D-penicillamine and dimercaprol exhibited a weak ability to move ^225^Ac from its origin, and most of the radioactivity remained at the origin. For the remaining chelators, 30–70% of the radioactivity was found between the origin and front of the chromatogram.

### 3.2. Biodistribution and Dosimetry of ^225^Ac in Mice

**Figure 4:**
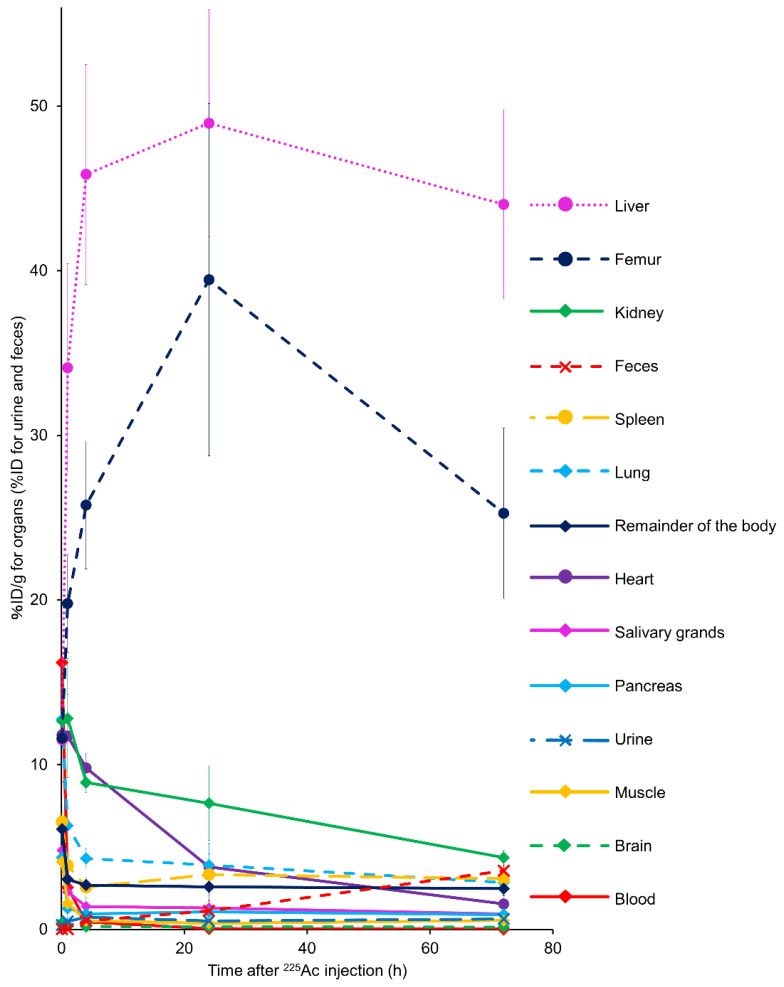
Biodistribution of free actinium-225 (^225^Ac) in BALB/c mice. Data were obtained at 5 min, 1 h, 4 h, 24 h, and 72 h after intravenous ^225^Ac injection. Values are expressed as % ID/g for the organs and blood and as the % ID for urine and feces. Values are shown as the mean ± SD (*n* = 4).

Time–activity curves for the collected organs and urinary and fecal excretions are shown in Figure 4. A noticeable ^225^Ac accumulation was observed in the liver, followed by the bones, throughout the observation period. A small amount of ^225^Ac, less than 5% of the total dose at 72 h after administration, was excreted in feces. Additionally, a negligible amount of ^225^Ac was excreted through the urine in mice. These results show a low whole-body clearance of free ^225^Ac in mice. The mean absorbed doses of ^225^Ac (mSv/MBq) in humans were estimated based on the biodistribution data from the mice (Table 2). The liver and bones showed relatively high estimated human absorbed doses.

### 3.3. Effect of Chelator Administration In Vivo

#### 3.3.1. Experiment 1

**Figure 5:**
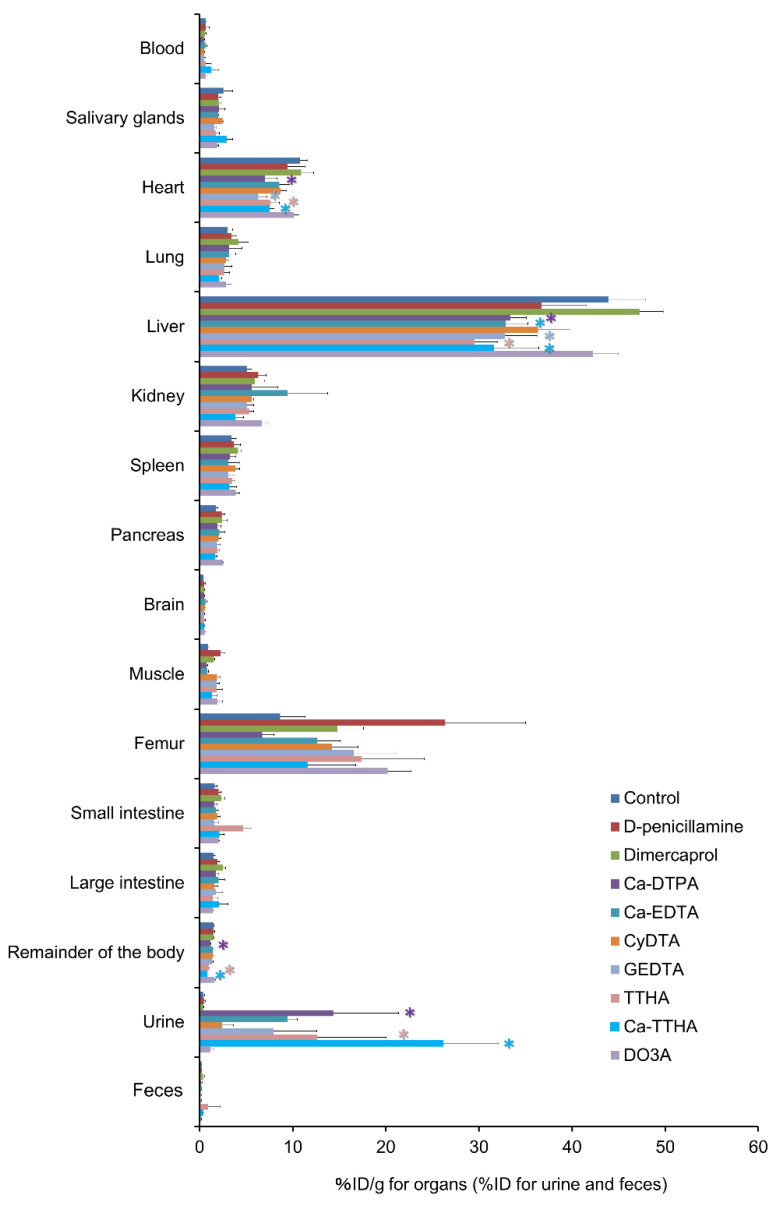
The effect of chelator administration on the biodistribution of free actinium-225 (^225^Ac). * indicates statistical significance (*p* < 0.05, vs. control).

Figure 5 shows differences in the biodistribution of ^225^Ac activities at 4 h, following ^225^Ac injection between the control and chelator groups. Ca-DTPA, Ca-EDTA, GEDTA, TTHA, and Ca-TTHA showed significant reductions in liver uptake of ^225^Ac (*p* < 0.05). There were significant reductions in ^225^Ac with Ca-DTPA, GEDTA, TTHA, and Ca-TTHA in the heart, and with Ca-DTPA, TTHA, and Ca-TTHA in the remainder of the body (*p* < 0.05). Urinary excretion of ^225^Ac was significantly accelerated with Ca-DTPA, TTHA, and Ca-TTHA (*p* < 0.05). There was a moderate positive correlation between the levels of chelator interactions with free ^225^Ac in vitro and ^225^Ac reduction in the liver in vivo on scatter plot analysis (*R*^2^ = 0.526, *p* < 0.05) (Supplementary Material Figure S1).

Biodistribution of ^225^Ac in the control and chelator groups. Mice were euthanized 4 h after the ^225^Ac injection. Values are shown as means ± SD; *n* = 4–5 (see details in the Methods section). * indicates statistical significance (*p* < 0.05, vs. control).

#### 3.3.2. Experiment 2

**Figure 6:**
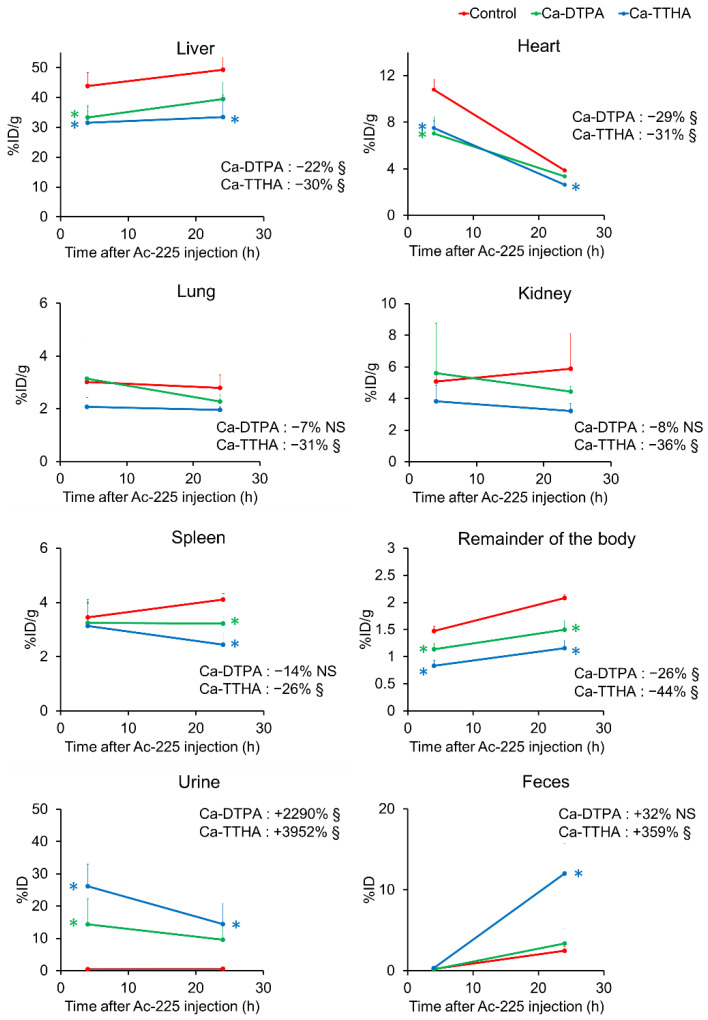
Time–activity curves of free actinium-225 (^225^Ac) after Ca-DTPA and Ca-TTHA administration in major organs, urine, and feces. Time–activity curves were generated using the biodistribution of ^225^Ac in control mice and mice with Ca-DTPA and Ca-TTHA at 4 and 24 h after ^225^Ac injection. Values are shown as mean ± SD; *n* = 4–5. Numbers in the graphs show the % increase (positive) or decrease (negative) of the area-under-the-curve in each chelator group, compared to the control. § indicates the statistical significance of time–activity curves (*p* < 0.05, vs. control). NS = not significant. * indicates statistical significance at each time point (*p* < 0.05, vs. control). The data for the other organs are shown in Figure S2.

Ca-DTPA and Ca-THHA were selected from among the other chelators to observe their effects over a longer period, as they showed a higher reduction rate for ^225^Ac liver uptake and urine excretion. At 1 h post ^225^Ac administration, Ca-DTPA and Ca-THHA were administered to the mice, as performed in Experiment 1, and the mice were euthanized 24 h after the ^225^Ac injection. Figure 6 and Supplementary Material Figure S2 show the time–activity curves of the selected organs of the control and chelator groups. The time–activity curve analysis show that the liver, heart, and remainder of the body exhibited significant ^225^Ac reductions in the Ca-DTPA and Ca-TTHA groups, and the lung, kidney, spleen, and pancreas showed significant ^225^Ac reductions in the Ca-TTHA group (*p* < 0.05, vs. control). The reduction in the area-under-the-curve of the liver was 22% with Ca-DTPA and 30% with Ca-TTHA (Figure 6 and Supplementary Material Figure S2). For the femur, ^225^Ac retention was reduced, although not significantly. Ca-DTPA and Ca-TTHA showed significant increases in urinary excretion of ^225^Ac, and Ca-TTHA showed a significant increase in the fecal excretion of ^225^Ac (*p* < 0.05 vs. control), but Ca-DTPA did not exhibit any significant increase in ^225^Ac excretion.

## 4. Discussion

In this study, we demonstrated that aminopolycarboxylate chelators with five and six carboxylic groups, Ca-DTPA and Ca-TTHA, respectively, induced whole-body clearance of free ^225^Ac, with a significant reduction in the liver as the critical organ. ^225^Ac was excreted in the urine and feces after chelation. The liver showed the highest retention of free ^225^Ac with an estimated human absorbed dose of 4.76 Sv_RBE5_/MBq. Therefore, Ca-DTPA and Ca-TTHA administration may be a treatment option for unexpected radiation exposure caused by free ^225^Ac.

Aminopolycarboxylate chelators, such as DTPA, EDTA, GEDTA, and TTHA, showed higher reductions in ^225^Ac retention in the liver than D-penicillamine, dimercaprol, and DO3A. These results reflect differences in the number of ligating atoms and the number of coordinating groups. The Ac^3+^ ion is classified as a ‘hard’ Lewis acid, according to the hard and soft acids and bases (HSAB) theory [34], as it behaves similar to the La^3+^ ion, carrying a large charge and low polarizability. Thus, the Ac^3+^ ion prefers hard bases or non-polarizable and negatively charged Lewis bases such as carboxylates [35], and thus shows a high affinity for aminopolycarboxylates, especially those with a higher number of carboxylic groups, such as DTPA and TTHA. D-penicillamine and dimercaprol are sulfur-coordinating soft Lewis bases that are effective decorporation chelators for soft metal ions, such as Pb^2+^, Hg^2+^, and As^3+^.

The aminopolycarboxylates used in this study behave as multidentate chelators for the Ac^3+^ ion. The thermodynamic stability increases with the number of coordinating atoms [21]. Indeed, our results are consistent with the stability constants for La^3+^ complexes [36]. The low decorporation ability of DO3A can be explained similarly. Since fast reaction rates are characteristic of metal complex formation and the exchange reaction of open-chain polyaminocarboxylates [37], the above qualitative thermodynamic discussion would be applicable. In contrast, the low reactivity of CyDTA may be observed because of its kinetic inertness. The kinetic inertness of CyDTA is well-recognized and is induced by the rigidity of the cyclohexyl bridge [38,39]. The same description may also be relevant to DO3A to some extent.

Ca-TTHA and the sodium salt of TTHA exhibited similar effects on the ^225^Ac distribution pattern in mice. Previous studies using EDTA have reported that its calcium salt, rather than sodium salt, is preferred as a chelator drug, because Na-EDTA chelates Ca in the body and may cause hypocalcemic tetany [40]. Therefore, Ca-TTHA should be selected for the development of TTHA as a chelator drug.

In this study, we administered a series of chelators 1 h following ^225^Ac injection. The chelator administration time was selected based on the distribution data, which indicated that most of the free ^225^Ac was distributed in the liver. We found that Ca-DTPA and Ca-TTHA reduced free ^225^Ac retention in the liver, as well as in other organs. These results show that Ca-DTPA and Ca-TTHA are effective for reducing radiation exposure from free ^225^Ac. Ca-TTHA showed a higher tendency to reduce ^225^Ac retention than Ca-DTPA in the various organs. These data support the development of Ca-TTHA for the removal of free ^225^Ac. To produce ^225^Ac for medical use, ^229^Th generators are currently used, while accelerator synthesis of ^225^Ac is under investigation to increase the supply [41]. In accelerator synthesis, ^227^Ac (*T*_1/2_ = 21.8 y), which cannot be chemically separated, is included as an unavoidable by-product [41]. The method proposed by the present study may, thus, also be useful to reduce this ^227^Ac, which will be retained in the liver for substantially longer than ^225^Ac in ^225^Ac TAT. Aminopolycarboxylates are known to form stable chelates with a wide range of metal ions. DTPA and TTHA can be expected to capture the daughter nuclide ^213^Bi (and to a lesser extent, ^209^Pb^2+^) de-chelated by the recoil during the decay process of ^225^Ac, as these two chelators form highly stable complexes with Bi^3+^ [36].

This study had several limitations. First, free ^225^Ac was evaluated in this study since free ^225^Ac can be released from the ^225^Ac-labeled drugs and could cause unexpected toxicity in ^225^Ac TAT in general. Since the biodistribution and kinetics are dependent on each drug, further preclinical and clinical studies that specifically target ^225^Ac-labeled drugs are necessary to carefully determine the appropriate use of the method developed in this study, with consideration of their specific pharmacokinetic properties. Second, we used non-tumor-bearing mice in this study. In order not to affect tumor uptake of ^225^Ac-labeled drugs, it is necessary to investigate the timing of chelator administration for each ^225^Ac-labeled drug using tumor-bearing mice. The ^225^Ac internalization by tumor cells following drug delivery is important in drug design in the development of agents for ^225^Ac TAT, because internalization causes the short-lived daughter radionuclides generated by ^225^Ac to be trapped in the cells [42,43]. Therefore, chelator administration timing would be appropriate after tumor delivery and internalization because chelators with negatively charged carboxylic groups do not penetrate tumor cell membranes [44]. Finally, this study used a fixed administration dose and the same administration route for aminopolycarboxylate chelators (150 mg/kg, i.p.) for comparisons. Optimization of the administration dose and route and safety tests should be addressed in future studies.

## 5. Conclusions

We found that aminopolycarboxylate chelators with five and six carboxyl groups, Ca-DTPA and Ca-TTHA, are useful for the whole-body clearance of free ^225^Ac; with a remarkable reduction in the liver. Our findings provide a novel strategy for removing accumulated free ^225^Ac released from ^225^Ac-labeled drugs and encourage the future development of ^225^Ac TAT.

## Figures and Tables

**Table 1:** Candidate chelators to reduce free actinium-225 (^225^Ac) (dose, route, and information).

Chelators	Abbreviations	Providers	Dose	Administration Volume and Route
D-Penicillamine	-	Fujifilm Wako Chemicals	300 mg/kg	150 µL p.o.
2,3-Dimercapto-1-propanol	Dimercaprol	Tokyo Chemical Industries	60 mg/kg	50 µL i.m.
Calcium diethylenetriamine-*N*,*N*,*N*′,*N*″,*N*″-pentaacetate	Ca-DTPA	Chemisch-pharmazeutische Fabrik GmbH	150 mg/kg	100 µL i.p.
Calcium ethylenediamine-*N*,*N*,*N*′,*N*′-tetraacetate	Ca-EDTA	Fujifilm Wako Chemicals	150 mg/kg	100 µL i.p.
*trans*-1,2-Diaminocyclohexane-*N*,*N*,*N*′,*N*′-tetraacetic acid	CyDTA	Fujifilm Wako Chemicals	150 mg/kg	100 µL i.p.
*O*,*O*’-Bis(2-aminoethyl)ethyleneglycol-*N*,*N*,*N*′,*N*′-tetraacetic acid	GEDTA	Fujifilm Wako Chemicals	150 mg/kg	100 µL i.p.
Triethylenetetramine-*N*,*N*,*N*′,*N*″,*N*‴,*N*‴-hexaacetic acid	TTHA	Fujifilm Wako Chemicals	150 mg/kg	100 µL i.p.
Calcium triethylenetetramine-*N*,*N*,*N*′,*N*″,*N*‴,*N*‴-hexaacetate	Ca-TTHA	See text	150 mg/kg	100 µL i.p.
1,4,7,10-Tetraazacyclododecane-1,4,7-triacetic acid	DO3A	Macrocyclics	150 mg/kg	100 µL i.p.

**Table 2:** Mean estimated human absorbed doses for free actinium-225 (^225^Ac), extrapolated from mice biodistribution data.

	Estimated Absorbed Dose (mSv_RBE5_/MBq)	
Target Organ	Male	Female
Adrenals	2.34 × 10^2^	3.03 × 10^2^
Brain	5.62	6.66
Breasts	2.34 × 10^2^	3.03 × 10^2^
Gallbladder wall	2.34 × 10^2^	3.03 × 10^2^
Lower large intestinal wall	2.34 × 10^2^	3.03 × 10^2^
Small intestine	2.34 × 10^2^	3.03 × 10^2^
Stomach wall	2.34 × 10^2^	3.03 × 10^2^
Upper large intestinal wall	2.34 × 10^2^	3.03 × 10^2^
Heart wall	3.85 × 10^2^	5.06 × 10^2^
Kidneys	2.15 × 10^2^	2.33 × 10^2^
Liver	4.76 × 10^3^	6.50 × 10^3^
Lungs	1.61 × 10^2^	2.01 × 10^2^
Muscle	2.86 × 10^1^	4.70 × 10^1^
Ovaries	2.34 × 10^2^	3.03 × 10^2^
Pancreas	4.79 × 10^1^	5.33 × 10^1^
Red marrow	3.37 × 10^2^	3.89 × 10^2^
Osteogenic cells	1.17 × 10^4^	1.63 × 10^4^
Skin	2.34 × 10^2^	3.03 × 10^2^
Spleen	3.79 × 10^2^	4.62 × 10^2^
Testes	2.34 × 10^2^	
Thymus	2.34 × 10^2^	3.03 × 10^2^
Thyroid	2.34 × 10^2^	3.03 × 10^2^
Urinary bladder wall	2.36 × 10^2^	3.05 × 10^2^
Uterus	2.34 × 10^2^	3.03 × 10^2^
Total body	4.45 × 10^2^	5.78 × 10^2^
Effective dose equivalent	8.72 × 10^2^	1.17 × 10^3^
Effective dose	5.64 × 10^2^	7.52 × 10^2^

## Data Availability

Data are within the article and Supplementary materials.

## Supplementary Materials

The following are available online at https://www.mdpi.com/article/10.3390/pharmaceutics13101706/s1, Figure S1: Comparison of chelator interactions with free ^225^Ac in vitro and in vivo, Figure S2: Time–activity curves of free ^225^Ac following Ca-DTPA and Ca-TTHA administration in the other organs.
