# Supplementary material for: Evaluation of Aminopolycarboxylate Chelators for Whole-Body Clearance of Free 225Ac: A Feasibility Study to Reduce Unexpected Radiation Exposure during Targeted Alpha Therapy
Source: Pharmaceutics. 2021 Oct 16;13(10):1706. doi: 10.3390/pharmaceutics13101706 (PMC8540721; doi:10.3390/pharmaceutics13101706)
Supplement: Supplementary file 1 [file pharmaceutics-13-01706-s001.zip › pharmaceutics-1394486-supplementary.pdf]

pharmaceutics

Supplementary Materials: Evaluation of Aminopolycarboxylate Chelators for Whole-Body Clearance of Free ^{225}Ac: A Feasibility Study to Reduce Unexpected Radiation Exposure during Targeted Alpha Therapy

Mitsuyoshi Yoshimoto, Yukie Yoshii, Hiroki Matsumoto, Mitsuhiro Shinada, Masashi Takahashi, Chika Igarashi, Fukiko Hihara, Tomoko Tachibana, Ayano Doi, Tatsuya Higashi, Hirofumi Fujii, Kohshin Washiyama

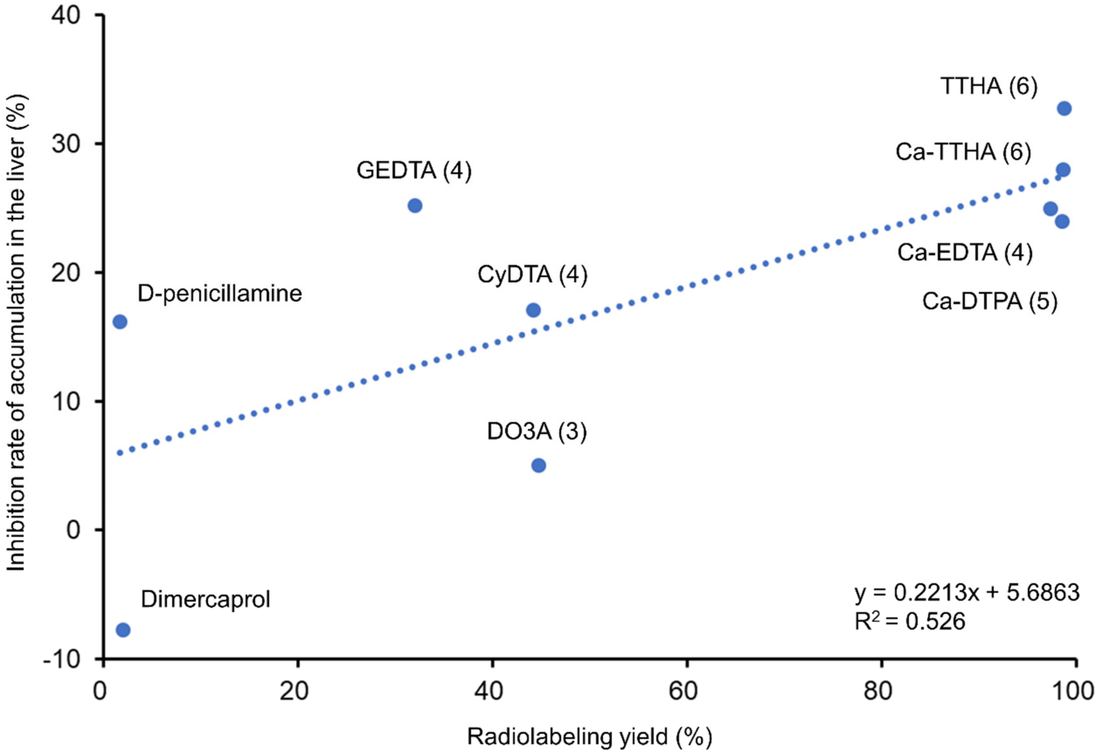

Figure S1. Comparison of chelator interactions with free ^{225}Ac in vitro and in vivo.Scatter plot showing a moderate positive correlation between the levels of chelator interaction with free ^{225}Ac in vitro and ^{225}Ac reduction in the liver in vivo ($R^2 = 0.526$, $P < 0.05$).

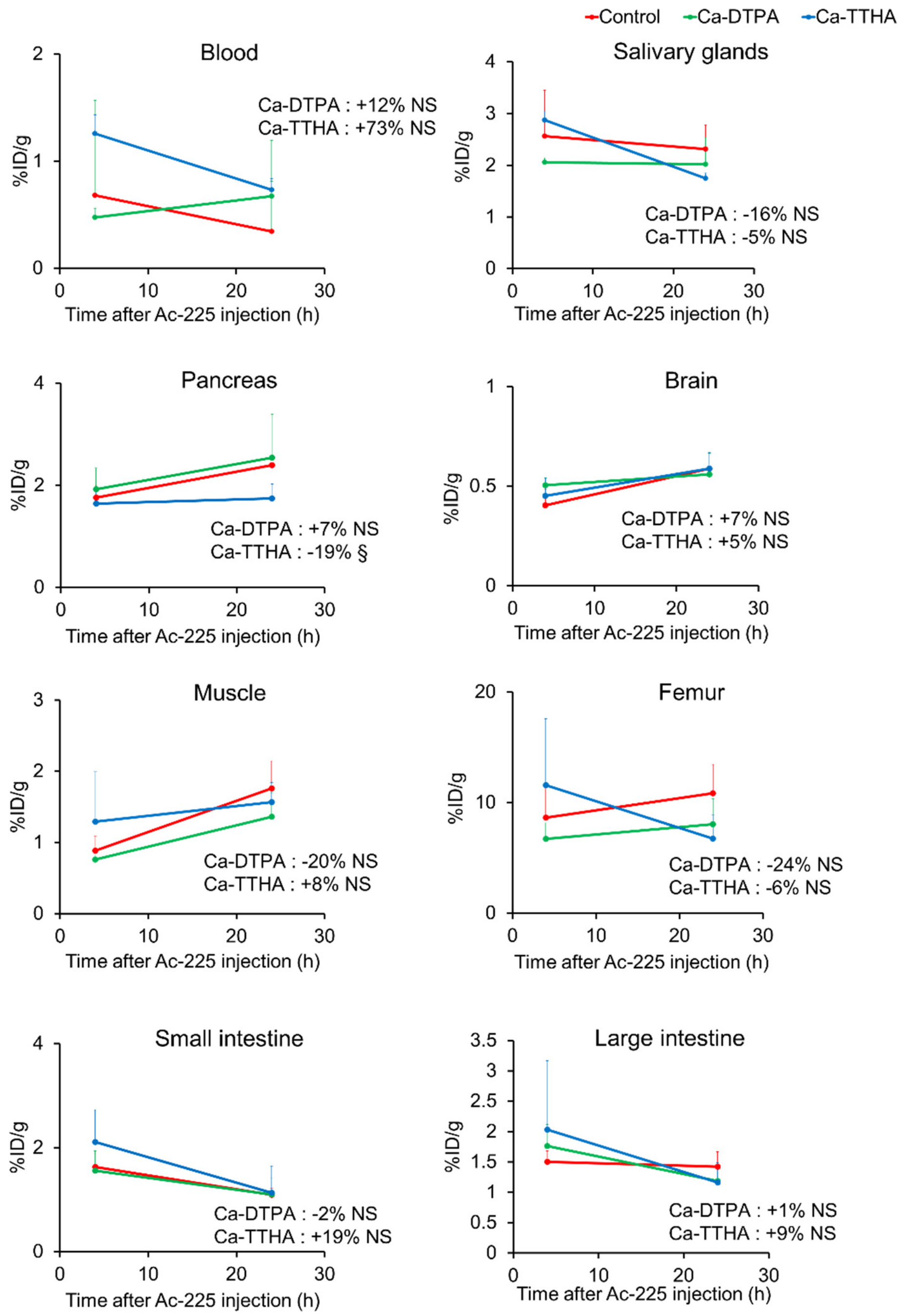

Figure S2. Time-activity curves of free ^{225}Ac following Ca-DTPA and Ca-TTHA administration in the other organs. Time activity curves of the other organs, which are not shown in Fig. 6, in the control and chelator groups. Time-activity curves were generated using the biodistribution of ^{225}Ac in control mice and mice with Ca-DTPA and Ca-TTHA at 4 and 24 h following ^{225}Ac injection. Values are shown as the means ± SD; n = 4–5. Numbers in the graphs show % increase (positive) or decrease (negative) of the area-under-the-curve in the chelating ligand groups, compared to the control. § Indicates statistical significance of time-activity curves ($P < 0.05$, vs. control). NS = not significant. There was no statistically significant difference at any time point in the organs in this figure.
